# Prevalence and factors associated with burnout syndrome among anesthesiology residents in Rio Grande do Sul, Brazil: a multicenter cross-sectional study

**DOI:** 10.1016/j.bjane.2026.844771

**Published:** 2026-06-01

**Authors:** Airton Bagatini, Felipe de Sousa Junqueira, Eduarda Druck Magadan, Silvia Ramos Hecktheuer, Regis Borges Aquino, Florentino Fernandes Mendes, Eduardo Francisco Mafassioli Corrêa, Cleiton da Silva Pando, Daniela Benzano Bumaguin, Cassiana Gil Prates

**Affiliations:** a Sociedade de Anestesiologia (SANE), Centro de Ensino e Treinamento, Porto Alegre, RS, Brazil; b Associação Hospitalar Beneficente São Vicente de Paulo, Centro de Ensino e Treinamento, Passo Fundo, RS, Brazil; c Pontifícia Universidade Católica do Rio Grande do Sul (PUC-RS), Hospital São Lucas, Centro de Ensino e Treinamento, Porto Alegre, RS, Brazil; d Universidade Federal de Ciências da Saúde de Porto Alegre (UFCSPA), Irmandade Santa Casa de Misericórdia de Porto Alegre, Centro de Ensino e Treinamento, Porto Alegre, RS, Brazil; e Hospital Universitário, Universidade de Santa Maria (UFSM), Centro de Ensino e Treinamento Professor Manoel Alvarez, Santa Maria, RS, Brazil; f Hospital de Clínicas de Porto Alegre (HCPA), Serviço de Anestesia e Medicina Perioperatória (SAMPE), Centro de Ensino e Treinamento, Porto Alegre, RS, Brazil; g Universidade Federal do Rio Grande do Sul (UFRGS), Centro de Pesquisa em Álcool e Drogas, Porto Alegre, RS, Brazil

**Keywords:** Anesthesiology, Burnout, Professional, Education, Medical, Graduate, Internship and Residency, Mental Health, Occupational Stress

## Abstract

**Background:**

Burnout syndrome is characterized by emotional exhaustion, depersonalization, and low personal accomplishment. Anesthesiology residents are particularly vulnerable due to prolonged working hours, cognitive overload, and exposure to stressful clinical situations. This study aimed to estimate the prevalence of burnout and explore associated occupational and psychosocial factors among anesthesiology residents in Rio Grande do Sul, Brazil.

**Methods:**

This multicenter cross-sectional study included residents from 6 training centers accredited by the Brazilian Society of Anesthesiology. Data were collected using an anonymous online questionnaire and the Portuguese-validated Maslach Burnout Inventory-Human Services Survey (MBI-HSS). Burnout was defined by high emotional exhaustion (≥ 25), high depersonalization (≥ 10), and low personal accomplishment (≤ 32). Associations were analyzed using Fisher’s exact test (p < 0.05).

**Results:**

Ninety-two residents participated (response rate, 84.4%). The overall prevalence of burnout was 26.1% (95% CI 17.5%–36.3%). High emotional exhaustion occurred in 72.8%, depersonalization in 42.4%, and low personal accomplishment in 43.5%. Burnout was significantly associated with living alone (p *=* 0.036) and psychotropic medication use (p = 0.017). A weekly workload of 60 hours or more (p = 0.068) was not significantly associated with burnout. Female residents showed higher emotional exhaustion (p = 0.039).

**Conclusion:**

Burnout prevalence among anesthesiology residents in Rio Grande do Sul was high, predominantly characterized by emotional exhaustion. Institutional strategies focusing on psychosocial support, workload management, and mental health promotion may improve residents’ well-being and professional performance.

## Introduction

Burnout syndrome was first described by Freudenberger in 1974 as a state of work-related physical and Emotional Exhaustion (EE) among healthcare professionals, characterized by a loss of energy and motivation as well as reduced effectiveness at work.^1^ Subsequently, Maslach and Jackson conceptualized burnout as a multidimensional construct comprising EE, Depersonalization (DP), and reduced Personal Accomplishment (PA), and developed the Maslach Burnout Inventory (MBI), which has since become the most widely used instrument for assessing this phenomenon.^2^^,^^3^ In 2019, the World Health Organization included burnout in the 11^th^ Revision of the International Classification of Diseases as an occupational phenomenon, recognizing it as the result of chronic, unmanaged workplace stress.^4^

Burnout prevalence estimates vary substantially depending on the definition and cutoff criteria used, particularly regarding whether one, two, or all three MBI dimensions are required for diagnosis. Studies adopting less stringent definitions, such as high scores in a single dimension, tend to report higher prevalence rates, whereas more conservative tridimensional definitions provide greater specificity but lower estimates. In the present study, we adopted a conservative tridimensional definition (high emotional exhaustion, high depersonalization, and low personal accomplishment) to improve the specificity and comparability of our findings.

Among healthcare professionals, burnout has emerged as a global threat to quality of care and patient safety. Evidence from systematic reviews indicates prevalence rates exceeding 50% in certain medical specialties, with consistent associations with medical errors, reduced productivity, and adverse outcomes.^5^^,^^6^ Its impact extends beyond the individual, adversely affecting healthcare teams, institutions, and the sustainability of health systems.^7^

Resident physicians represent a particularly vulnerable group. Multicenter studies report prevalence rates ranging from 30% to 60%, depending on specialty, year of training, and assessment methodology.^8^^,^^9^ Frequently cited risk factors include prolonged working hours, sleep deprivation, cognitive overload, repeated exposure to human suffering, institutional pressures, and insufficient social support.^9^^,^^10^ Consequences include increased risk of depression, suicidal ideation, attrition from the profession, and impaired clinical performance.^11^^,^^12^

In anesthesiology, these risks are further intensified by the need for continuous vigilance, rapid decision-making in critical situations, and management of highly complex clinical scenarios. Such characteristics heighten vulnerability to EE, placing anesthesiology residents at particularly high risk.^9^^,^^11^ Studies conducted in different countries consistently demonstrate high rates of burnout in this population, often exceeding those observed in other medical specialties.^12^^,^^13^

In Brazil, although burnout has been investigated in some medical specialties, data on anesthesiology residents remain limited. A national multicenter study among intensivists reported high prevalence rates,^14^ and research involving multiprofessional residents also revealed concerning levels of burnout.^15^ However, specific data on anesthesiology residents in Rio Grande do Sul, the southernmost state of Brazil ‒ an area with a strong tradition in medical education and a substantial number of training centers accredited by the Brazilian Society of Anesthesiology (SBA) ‒ are lacking. This gap hinders the development of institutional strategies tailored to the local context and the implementation of effective prevention and support policies.

We hypothesized that anesthesiology residents training in SBA-accredited centers in Rio Grande do Sul, Brazil, have a high prevalence of burnout syndrome and that its occurrence is associated with occupational and psychosocial factors, including workload, sleep deprivation, and perceived institutional support. Therefore, this study aimed to estimate the prevalence of burnout and explore associated occupational and psychosocial factors among anesthesiology residents training in SBA-accredited centers in Rio Grande do Sul, Brazil.

## Methods

### Study design

This multicenter cross-sectional study included all 6 SBA-accredited training centers in the state of Rio Grande do Sul, Brazil. The project was approved by the Institutional Review Board, and research and methods adhered to the provisions of the Declaration of Helsinki and the Strengthening the Reporting of Observational Studies in Epidemiology (STROBE) guidelines.

### Ethical aspects

The study protocol was approved by the Research Ethics Committee of Hospital Ernesto Dornelles, Porto Alegre, RS, Brazil (approval number: 7.337.285; CAAE 85752324.2.0000.5304), in accordance with Resolution Number 466/2012 of the Brazilian National Health Council. Data collection was conducted anonymously using an electronic questionnaire (Google Forms). Access to the survey questions was preceded by mandatory reading and electronic acceptance of the Informed Consent Form (ICF). Acceptance of the ICF (by clicking “I agree”) enabled the resident to proceed with the questionnaire, constituting formal informed consent prior to participation. Data confidentiality and participant anonymity were strictly preserved throughout all study phases.

### Study population and eligibility

The target population comprised 109 anesthesiology residents eligible across the 6 SBA-accredited training centers in Rio Grande do Sul. Participant recruitment occurred between July and August 2025, and all residents were invited via electronic message containing a link to the online questionnaire.

Inclusion criteria were enrollment in the first, second, or third year of anesthesiology residency at an SBA-accredited center in Rio Grande do Sul and electronic acceptance of the ICF, which was required to access and complete the questionnaire. Exclusion criteria included residents who were on leave at the time of data collection (medical leave, maternity leave, vacation, or other reasons), as well as those who declined to provide electronic informed consent despite invitation and clarification of the study protocol.

Electronic questionnaires with incomplete data, defined as missing responses in more than 10% of the MBI-HSS items or in key sociodemographic variables, would be excluded to preserve the validity of burnout assessment. A total of 109 residents were invited to participate, of whom 92 responded. No participants met exclusion criteria, and all respondents were included in the final analysis.

### Data collection and instruments

Data were collected using an anonymous, self-administered, and self-explanatory electronic questionnaire developed on the Google Forms platform. The instrument was divided into 2 main sections: 1) Sociodemographic and occupational variables, and 2) Assessment of burnout syndrome.

The first section consisted of a structured questionnaire designed to capture potential risk factors for burnout. Variables included resident characteristics such as sex, age group, marital status, living arrangement (living alone), year of residency, weekly workload, engagement in external professional activities, motivation for choosing anesthesiology, lifestyle habits (physical activity and alcohol consumption), and mental health-related factors (use of psychotropic medications and engagement in psychotherapy).

The second section assessed burnout using the MBI-Human Services Survey (MBI-HSS), in its Portuguese-language validated version.^16^ The MBI-HSS is considered the gold standard for burnout assessment and consists of 22 items distributed across 3 core dimensions: EE (9 items), DP (5 items), and PA (8 items). Each item is rated on a 7-point Likert scale ranging from 0 (“never”) to 6 (“every day”), reflecting the frequency of work-related feelings. Each MBI-HSS dimension (emotional exhaustion, depersonalization, and personal accomplishment) was analyzed separately, with scores calculated according to standard procedures for each subscale.

Items related to EE (items 1, 2, 3, 6, 8, 13, 14, 16, and 20) and DP (items 5, 10, 11, 15, and 22) are negatively oriented, with higher scores indicating greater burnout severity. In contrast, items assessing PA (items 4, 7, 9, 12, 17, 18, 19, and 21) are positively oriented, with higher scores reflecting lower burnout levels. Burnout dimensions were analyzed separately according to established cutoff points. Overall burnout syndrome was defined by the simultaneous presence of high EE, high DP, and low PA, in accordance with validated tridimensional criteria.

### Definition of burnout syndrome

Burnout syndrome classification and prevalence were determined using tridimensional cutoff points established in national and international validation studies and systematic reviews based on MBI-HSS scores. Subscale scores were categorized as high (presence) or low (absence) burnout according to the following thresholds: high EE (≥ 25), high DP (≥ 10), and low PA (≤ 32). The simultaneous presence of high EE, high DP, and low PA was used to define overall burnout syndrome.^5^^,^^11^

These cutoff points are based on validation studies of the Portuguese version of the MBI-HSS and have been widely adopted in Brazilian studies involving healthcare professionals. Their use enhances the cultural and linguistic validity of the assessment in this population and allows for meaningful comparison with national and international literature.

### Sample size calculation

Sample size was calculated using WINPEPI, version 11.65. The calculation was based on the total number of anesthesiology residents accredited in SBA training centers as of June 30, 2025 (n = 109), assuming an expected burnout prevalence of 51% (based on prior literature),^8^ a 95% confidence level, and a margin of error of 10 percentage points. Accounting for an estimated 10% rate of losses and refusals, the minimum required sample size was 59 respondents to ensure the minimum sample size required to achieve the desired precision for prevalence estimation.

### Statistical analysis

Participant responses, initially recorded in Google Sheets, were compiled and exported to IBM SPSS, version 20.0, for statistical analysis. Burnout prevalence was reported with corresponding 95% Confidence Intervals (95% CI). Categorical variables were described using absolute (n) and relative (%) frequencies. Associations between burnout syndrome (and its dimensions) and sociodemographic and occupational variables were assessed using Fisher’s exact test. A two-tailed p < 0.05 was considered statistically significant for all analyses. Participants were nested within six training centers. Although center-level characteristics may influence both exposures and burnout, the analyses were conducted assuming independence between observations, given the similar structure and training conditions across centers.

Given the exploratory nature of the study, no formal adjustment for multiple comparisons was performed. The analyses of factors associated with burnout were secondary and exploratory. Considering the sample size and the limited number of outcome events, multivariable modeling was not performed due to the risk of overfitting and unstable estimates. In addition, considering the rule-of-thumb of at least 10 events per variable, the number of burnout cases would not support a stable multivariable model including multiple covariates. Therefore, only bivariate analyses were conducted, and the findings should be interpreted as exploratory associations rather than independent effects.

## Results

A total of 92 anesthesiology residents from the 6 SBA-accredited training centers in Rio Grande do Sul participated in the study, corresponding to a response rate of 84.4% (92/109). The sample consisted predominantly of men (56.5%), with most participants aged 25 to 30 years (54.3%) and a median age of 29 years. The majority were in a stable relationship or dating (66.3%), and nearly half lived alone (47.8%). Residency year distribution was balanced: 31.5% were first-year, 32.6% second year, and 35.9% third-year residents.

Weekly workload was high, with 65.2% of residents reporting ≥ 60 working hours per week and 33.7% reporting 40‒60 hours. Nearly half (48.9%) reported engaging in professional activities outside the residency program, and 45.7% stated that anesthesiology was their first-choice specialty. Regarding lifestyle factors, 52.2% had a pet, 64.2% engaged in regular physical activity (up to 4 times per week), and 41.3% reported psychotropic medication use. Approximately one-third (31.5%) were undergoing psychotherapy, and 48.9% reported alcohol consumption once or twice per week (Table 1).

**Table 1:** Sample characteristics (n = 92).

Characteristic	n (%)
Sex	
Male	52 (56.5)
Female	40 (43.5)
Age group	
20‒25 years	7 (7.6)
25‒30 years	50 (54.3)
30‒35 years	31 (33.7)
35‒40 years	4 (4.3)
Living alone	44 (47.8)
Marital status	
Married/stable relationship/dating	61 (66.3)
Single	31 (33.7)
Year of residency	
First year	29 (31.5)
Second year	30 (32.6)
Third year	33 (35.9)
Weekly workload	
20‒40 hours	1 (1.1)
40‒60 hours	31 (33.7)
60 or more hours	60 (65.2)
Hold jobs outside the residency program	45 (48.9)
Anesthesiology as first-choice specialty	42 (45.7)
Pet ownership	48 (52.2)
Engagement in psychotherapy	29 (31.5)
Psychotropic medication use	38 (41.3)
Alcohol consumption	
No	40 (43.5)
Once or twice per week	45 (48.9)
More than twice per week	7 (7.6)
Physical activity	
No	26 (28.2)
1 to 4 times per week	59 (64.2)
More than 4 times per week	7 (7.6)

The overall prevalence of burnout was 26.1% (95% CI 17.5%‒36.3%). When analyzed separately, the MBI-HSS dimensions showed high EE in 72.8% (95% CI 62.6%‒81.6%), high DP in 42.4% (95% CI 32.2%‒53.1%), and low PA in 43.5% (95% CI 33.2%‒54.2%). These findings indicate a high degree of emotional strain among residents, with substantial impairment across the other burnout dimensions (Table 2).

**Table 2:** Prevalence of burnout (n = 92).

Variable	% [95% CI]
Overall burnout	26.1 [17.5 – 36.3]
Emotional exhaustion	72.8 [62.6 – 81.6]
Depersonalization	42.4 [32.2 – 53.1]
Low personal accomplishment	43.5 [33.2 – 54.2]

CI, Confidence Interval.

Table 3 presents the analysis of factors potentially associated with burnout syndrome and its subdimensions. The category of 20–40 weekly hours was not included in comparative analyses due to insufficient sample size. Living alone was significantly associated with a higher overall prevalence of burnout (36.4% vs. 16.7%; p = 0.036; Prevalence Ratio: 2.18; 95% CI 1.04‒4.59). Among the burnout dimensions, female sex was associated with a higher frequency of EE (85.0% vs. 63.5%; p = 0.039; Prevalence Ratio = 1.34; 95% CI 1.05‒1.71), suggesting greater vulnerability of women to the emotional component of burnout.

**Table 3:** Factors potentially associated with overall burnout, Emotional Exhaustion (EE), Depersonalization (DP), and low Personal Accomplishment (PA).

aCharacteristic	Overall Burnout	p	EE	p	DP	p	PA	p
**Sex**		0.160		0.039		0.296		0.638
Male	17 (32.7%)		33 (63.5%)		25 (48.1%)		21 (40.4%)	
Female	7 (17.5%)		34 (85.0%)		14 (35.0%)		19 (47.5%)	
**Age group**		0.792		0.875		0.082		0.216
20–25 years	2 (28.6%)		5 (71.4%)		4 (57.1%)		3 (42.9%)	
25–30 years	15 (30.0%)		38 (76.0%)		26 (52.0%)		18 (36.0%)	
30–35 years	6 (19.4%)		21 (67.7%)		8 (25.8%)		18 (58.1%)	
35–40 years	1 (25.0%)		3 (75.0%)		1 (25.0%)		1 (25.0%)	
**Living alone**		0.036		0.999		0.206		0.141
Yes	16 (36.4%)		32 (72.7%)		22 (50.0%)		23 (52.3%)	
No	8 (16.7%)		35 (72.9%)		17 (35.4%)		17 (35.4%)	
**Marital status**		0.802		0.322		0.824		0.514
Married	15 (24.6%)		42 (68.9%)		25 (41.0%)		25 (41.0%)	
Single	9 (29.0%)		25 (80.6%)		14 (45.2%)		15 (48.4%)	
**Year of residency**		0.383		0.999		0.560		0.254
First year	5 (17.2%)		21 (72.4%)		12 (41.4%)		9 (31.0%)	
Second year	10 (33.3%)		22 (73.3%)		15 (50.0%)		14 (46.7%)	
Third year	9 (27.3%)		24 (72.7%)		12 (36.4%)		17 (51.5%)	
**Weekly workload**		0.068		0.011		0.092		0.046
20–40 hours^a^								
40–60 hours	4 (12.9%)		18 (58.1%)		9 (29.0%)		9 (29.0%)	
≥ 60 hours	20 (33.3%)		49 (81.7%)		30 (50.0%)		31 (51.7%)	
**Jobs outside program**		0.202		0.485		0.833		0.674
Yes	14 (31.1%)		31 (68.9%)		20 (44.4%)		21 (46.7%)	
No	10 (21.3%)		36 (76.6%)		19 (40.4%)		19 (40.4%)	
**First-choice specialty**		0.641		0.347		0.207		0.294
Yes	12 (28.6%)		33 (78.6%)		21 (50.0%)		21 (50.0%)	
No	12 (24.0%)		34 (68.0%)		18 (36.0%)		19 (38.0%)	
**Pet ownership**		0.999		0.359		0.531		0.834
Yes	13 (27.1%)		37 (77.1%)		22 (45.8%)		20 (41.7%)	
No	11 (25.0%)		30 (68.2%)		17 (38.6%)		20 (45.5%)	
**Psychotherapy**		0.306		0.208		0.013		0.651
Yes	10 (34.5%)		24 (82.8%)		18 (62.1%)		14 (48.3%)	
No	14 (22.2%)		43 (68.3%)		21 (33.3%)		26 (41.3%)	
**Psychotropic medication**		0.017		0.016		0.005		0.087
Yes	15 (39.5%)		33 (86.8%)		23 (60.5%)		21 (55.3%)	
No	9 (16.7%)		34 (63.0%)		16 (29.6%)		19 (35.2%)	
**Alcohol consumption**		0.578		0.630		0.164		0.398
No	10 (25.0%)		27 (67.5%)		14 (35.0%)		14 (35.0%)	
1–2 week	11 (24.4%)		34 (75.6%)		20 (44.4%)		22 (48.9%)	
> 2 week	3 (42.9%)		6 (85.7%)		5 (71.4%)		4 (57.1%)	
**Physical activity**		0.593		0.698		0.999		0.207
No	6 (23.1%)		20 (76.9%)		11 (42.3%)		15 (57.7%)	
1–4 week	15 (25.4%)		41 (69.5%)		25 (42.4%)		22 (37.3%)	
> 4 week	3 (42.9%)		6 (85.7%)		3 (42.9%)		3 (42.9%)	

Data are presented as n (%) and compared using Fisher’s exact test.

a Categories with insufficient sample size were not included in statistical comparisons.

Weekly workload was significantly associated with both high EE (p = 0.011; Prevalence Ratio = 1.45; 95% CI 1.05‒2.02) and low PA (p = 0.046; Prevalence Ratio = 1.84; 95% CI 1.00‒3.37). However, no statistically significant association was observed between workload ≥ 60 hours and overall burnout (p = 0.068).

Psychotropic medication use was significantly associated with overall burnout syndrome (p = 0.017; Prevalence Ratio = 2.37; 95% CI 1.16‒4.84), EE (p = 0.016; Prevalence Ratio = 1.38; 95% CI 1.09‒1.75), and DP (p = 0.005; Prevalence Ratio = 2.04; 95% CI 1.26‒3.32). These findings should be interpreted as markers of psychological distress rather than causal factors. Similarly, residents undergoing psychotherapy exhibited a higher prevalence of DP (p = 0.013; Prevalence Ratio = 1.86; 95% CI 1.19‒2.92), which may reflect treatment-seeking behavior among individuals experiencing greater emotional burden.

No statistically significant associations were observed between burnout and age, marital status, year of residency, physical activity, alcohol consumption, pet ownership, or anesthesiology as first-choice specialty.

## Discussion

This multicenter study, involving a high-response multicenter sample of anesthesiology residents in Rio Grande do Sul, Brazil, identified an overall prevalence of burnout of 26.1% using the strict diagnostic criteria requiring all 3 MBI-HSS subscales. This finding underscores the occupational vulnerability of this population and highlights the need for immediate attention from residency training programs.

The observed burnout prevalence of 26.1% is consistent with the average reported in studies of residents in high-demand medical specialties in Brazil, although it remains substantially higher than what is observed in the general population. Importantly, because this estimate was derived using the strictest definition (requiring the simultaneous presence of high EE, high DP, and low PA), it likely underestimates the broader extent of psychological distress. Indeed, high EE was present in 72.8% of residents, emerging as the dominant feature of burnout in this cohort.

The high prevalence of EE among anesthesiology residents reflects the intrinsic demands of training in this specialty, which requires continuous vigilance, rapid decision-making in critical situations, and sustained exposure to stressful environments. EE, as the primary dimension of burnout, is a sensitive marker of chronic psychological overload and has been consistently identified as the most prevalent component in meta-analyses of Brazilian and international resident populations.^5^^,^^16^

Associations observed with variables such as psychotropic medication use, psychotherapy, and living alone likely reflect underlying psychological distress and contextual vulnerability rather than causal relationships. These findings should be interpreted cautiously, as no multivariable adjustment was performed, and causal inferences cannot be established. In addition, reverse causality and bidirectional relationships cannot be excluded, particularly for variables related to mental health care utilization.

In this context, the sample consisted predominantly of young adults (aged 25–35 years) with a balanced distribution across residency years, situating these findings within the period of greatest training intensity, during which the literature consistently reports heightened vulnerability to chronic occupational stressors.^8^, ^9^, ^10^, ^11^, ^12^^,^^17^, ^18^, ^19^

The overall burnout prevalence observed in this study (26.1%) lies within the lower-to-mid range of estimates reported for residents and anesthesiologists, ranging widely from 20% to 60% depending on definitions, instruments, and cutoff points used.^5^^,^^9^^,^^17^, ^18^, ^19^, ^20^, ^21^ In large cohorts of Brazilian anesthesiologists assessed during the COVID-19 pandemic, overall burnout prevalence was reported at 19.6%, whereas more than 50% were classified as being “at high risk” when EE or DP were applied alone, illustrating how less stringent definitions can substantially inflate prevalence estimates.^22^ This discrepancy was addressed in a recent review documenting more than 140 different definitions of burnout, even among studies using the MBI to assess burnout, resulting in prevalence estimates ranging from 0% to 80.5%. Conversely, studies requiring the simultaneous presence of high EE, high DP, and low PA report a substantially lower prevalence of burnout than those based on isolated dimensions.^21^

Among residents, systematic reviews and multicenter studies report burnout prevalence ranging from 30% to 50%, with heterogeneity according to specialty, workload, and institutional context.^17^, ^18^, ^19^^,^^22^^,^^23^ Our findings align with a recent Thai study of medical residents demonstrating strong associations between working conditions, mental health, burnout, and medical errors, reinforcing the clinical and operational relevance of this outcome.^19^ In Brazil, although using heterogeneous instruments, studies of multiprofessional residents have also reported concerning levels of burnout and suggested organizational determinants (workload, resource availability, and supervision) similar to those identified in medical residency programs.^15^

Regarding the MBI dimensions, the predominance of EE (72.8%) is consistent with studies identifying EE as the most stable and central component of the burnout construct, whereas DP and PA exhibit lower internal consistency and greater cultural variability.^2^^,^^3^^,^^24^ A recent review by Guille and Sen.^21^ highlights the weak correlation among the 3 domains and questions the validity of rigid cutoffs or composite scores for defining “cases”, a perspective that contextualizes both the magnitude and the precision of our estimates.

The association between working ≥ 60 hours per week and both EE and PA reflects one of the most consistently reported factors associated with burnout in the literature ‒ excessive workload and sleep deprivation - consistently associated with higher levels of exhaustion, impaired performance, and safety concerns.^8^, ^9^, ^10^, ^11^, ^12^^,^^19^^,^^21^ The finding of higher EE among women (85.0% vs. 63.5%) is consistent with evidence suggesting greater female vulnerability to organizational stressors in environments characterized by high responsibility and limited autonomy, mediated by additional non-work-related burdens and institutional biases.^21^^,^^25^^,^^26^

The significant association between living alone and overall burnout highlights the critical role of social support networks and interpersonal connectedness as modulators of resilience and coping strategies in high-stress settings such as residency training. This finding aligns with contemporary emphasis on interventions that strengthen peer support and promote accessible, destigmatized mental health care within educational and training institutions.^6^^,^^7^^,^^20^^,^^21^

The strong association between psychotropic medication use and overall burnout (as well as EE and DP) likely reflects greater severity of psychological distress and/or the presence of comorbid anxiety and depressive disorders. This relationship is well documented in the literature, which recognizes substantial conceptual and dimensional overlap between burnout and clinical conditions such as anxiety and depression. In this context, psychotropic medication use functions as a marker of distress severity, reinforcing the urgency of providing accessible mental health care.^11^^,^^12^^,^^21^ Similarly, the higher prevalence of DP among residents undergoing psychotherapy likely reflects treatment-seeking in more severe cases (indication bias) and should not be interpreted as evidence of therapeutic ineffectiveness.

This study has limitations that warrant consideration. We used the Portuguese-validated MBI-HSS and a conservative definition of overall burnout (high EE + high DP + low PA), an approach that is more specific and aligned with recommendations aimed at reducing prevalence overestimation.^2^^,^^3^^,^^5^^,^^17^^,^^24^ Nevertheless, the historical heterogeneity of the construct and ongoing debate regarding cutoffs and subscale combinations remain epistemological limitations that affect comparability across studies.^21^ Also, the cross-sectional design precludes causal inferences, and self-reported data may be subject to recall and social desirability bias. Additionally, no multivariable analysis was performed, which limits the ability to control for potential confounding factors and to estimate independent associations. Considering the rule-of-thumb of at least 10 events per variable, the number of burnout cases (n ≈ 24) would not support a stable multivariable model including the proposed covariates. Therefore, the findings should be interpreted as exploratory associations rather than independent effects.

Participants were clustered within training centers, and center-level characteristics may have influenced both exposures and outcomes. Due to the limited number of centers, more advanced multilevel approaches were not feasible. Given the multiple comparisons performed across several outcomes and exposure variables, there is an increased risk of type I error (false-positive findings). Therefore, the observed associations should be interpreted cautiously, with greater emphasis placed on effect sizes and confidence intervals rather than isolated p-values.

Non-response bias should also be considered, as residents who did not participate may differ systematically from respondents. However, the high response rate (84.4%) reduces the likelihood of substantial bias.

Despite these limitations, this study has important strengths, including its multicenter design, high response rate, and use of a validated instrument with a conservative definition of burnout. These characteristics enhance the internal consistency and regional relevance of the findings.

Our results highlight 3 priority axes for action within anesthesiology residency programs: 1) Workload management (monitoring duty hours, implementing sustainable schedules, and mitigating sleep deprivation); 2) Structured institutional support (mentorship, environments free from harassment or mistreatment, and safe psychological support channels); and 3) Streamlined access to evidence-based mental health care and well-being strategies.^6^^,^^7^^,^^19^, ^20^, ^21^ A recent study of Brazilian anesthesiologists suggests that leisure time exceeding 5 hours per week may be a protective factor, offering an operationally actionable insight for local policy development.^22^

Longitudinal studies are needed to elucidate trajectories of EE, DP, and PA across first, second, and third years of residency and during the transition to independent practice, as well as to evaluate multilevel interventions (systemic and individual) using clinical and educational outcomes such as errors, performance, retention, quality, and safety.^6^^,^^9^^,^^19^^,^^21^ Efforts to standardize measurement (by reporting domains separately and avoiding non-validated composite scores) may further enhance international comparability and support the development of more precise policies.^21^^,^^24^

## Conclusion

The overall prevalence of burnout (26.1%) among anesthesiology residents in Rio Grande do Sul was high, with emotional exhaustion emerging as the predominant component. Living alone and psychotropic medication use were associated with burnout, the latter likely representing a marker of psychological distress rather than a causal factor. Given the cross-sectional design and exploratory analyses, these findings should be interpreted with caution. These results highlight the need for institutional policies focused on strengthening psychosocial support and promoting mental health throughout medical training.

## Conflicts of interest

The authors declare no conflicts of interest.

## Data Availability

The datasets generated and/or analyzed during the current study are available from the corresponding author upon reasonable request.

## References

[1] Freudenberger HJ. (1974). Staff burn-out. J Soc Issues.

[2] Maslach C., Jackson SE. (1981). The measurement of experienced burnout. J Organ Behav.

[3] Maslach C., Schaufeli W.B., Leiter MP. (2001). Job burnout. Annu Rev Psychol.

[4] World Health Organization (2019). Burn-out an “occupational phenomenon”: International Classification of Diseases [Internet]. https://www.who.int/news/item/28-05-2019-burn-out-an-occupational-phenomenon-international-classification-of-diseases.

[5] Rotenstein L.S., Torre M., Ramos M.A. (2018). Prevalence of burnout among physicians: a systematic review. JAMA.

[6] Panagioti M., Panagopoulou E., Bower P. (2017). Controlled interventions to reduce burnout in physicians: a systematic review and meta-analysis. JAMA Intern Med.

[7] Dzau V.J., Kirch D., Nasca T. (2020). Preventing a parallel pandemic ‒ A national strategy to protect clinicians’ well-being. N Engl J Med.

[8] Patel R.S., Bachu R., Adikey A., Malik M., Shah M. (2018). Factors related to physician burnout and its consequences: a review. Behav Sci (Basel).

[9] Sun H., Warner D.O., Macario A., Zhou Y., Culley D.J., Keegan MT. (2019). Repeated cross-sectional surveys of burnout, distress, and depression among anesthesiology residents and first-year graduates. Anesthesiology.

[10] De Hert S. (2020). Burnout in healthcare workers: prevalence, impact and preventative strategies. Local Reg Anesth.

[11] Bagatini A., Bumaguin D.B., Bagatini L.Z., Ardais AP. (2025). The relationship between burnout syndrome and emotional intelligence in anesthesiologists during the COVID-19 pandemic: a cross-sectional study. J Patient Saf Risk Manag.

[12] Dyrbye L.N., Shanafelt T.D., Sinsky C.A. (2017). Burnout among health care professionals: a call to explore and address this underrecognized threat to safe, high-quality care. NAM Perspect.

[13] Eckleberry-Hunt J., Kirkpatrick H., Barbera T. (2018). The problems with burnout research. Acad Med.

[14] Tironi M.O.S., Teles J.M.M., Barros D.D.S. (2016). Prevalence of burnout syndrome in intensivist doctors in five Brazilian capitals. Rev Bras Ter Intensiva.

[15] Mesquita V., Malagris L. (2020). Burnout syndrome in multiprofessional health residents of a university hospital. Rev SBPH.

[16] Tamayo M.R., Tróccoli BT. (2009). Construction and factorial validation of the Burnout Characterization Scale (ECB). Estud Psicol (Natal).

[17] Chong M.Y.F., Lin S.H.X., Lim W.Y., Ong J., Kam P.C.A., Ong SGK. (2022). Burnout in anaesthesiology residents: a systematic review of its prevalence and stressors. Eur J Anaesthesiol.

[18] Bui D., Winegarner A., Kendall M.C., Almeida M., Apruzzese P., De Oliveira GS. (2023). Burnout and depression among anesthesiology trainees in the United States: an updated national survey. J Clin Anesth.

[19] Surawattanasakul V., Siviroj P., Kiratipaisarl W. (2024). Resident physician burnout and association with working conditions, psychiatric determinants, and medical errors: a cross-sectional study. PLoS One.

[20] World Health Organization. Mental health and psychosocial considerations for health workers [Internet]. 2020 [cited 2025 Dec 16]. Available from: https://www.who.int/docs/default-source/coronaviruse/mental-health-considerations.pdf.

[21] Guille C., Sen S. (2024). Burnout, depression, and diminished well-being among physicians. N Engl J Med.

[22] Azi LMTA, Ferreira T.S., Cerqueira-Silva T., Diego L.A.S., Albuquerque M.A.C., Azi ML. (2025). Prevalence of burnout syndrome in Brazilian anesthesiologists during the COVID-19 pandemic: a cross-sectional survey. PLoS One.

[23] Rodrigues H., Cobucci R., Oliveira A. (2018). Burnout syndrome among medical residents: a systematic review and meta-analysis. PLoS One.

[24] Almeida G.D.C., Souza H.R.D., Almeida P.C.D., Almeida B.D.C., Almeida GH. (2016). The prevalence of burnout syndrome in medical students. Arch Clin Psychiatry (São Paulo).

[25] Maslach C., Jackson S.E., Leiter MP. (2018).

[26] Hu Y.Y., Ellis R.J., Hewitt D.B. (2019). Discrimination, abuse, harassment, and burnout in surgical residency training. N Engl J Med.

